# Salicylic acid treatment and expression of an *RNA-dependent RNA polymerase 1* transgene inhibit lethal symptoms and meristem invasion during tobacco mosaic virus infection in *Nicotiana benthamiana*

**DOI:** 10.1186/s12870-016-0705-8

**Published:** 2016-01-13

**Authors:** Wing-Sham Lee, Shih-Feng Fu, Zheng Li, Alex M. Murphy, Elizabeth A. Dobson, Laura Garland, Srinivasa Rao Chaluvadi, Mathew G. Lewsey, Richard S. Nelson, John P. Carr

**Affiliations:** Department of Plant Sciences, University of Cambridge, Downing Street, Cambridge, CB2 3EA UK; Plant Biology Division, Samuel Roberts Noble Foundation, Inc, 2510 Sam Noble Parkway, Ardmore, OK 73401 USA; Department of Biology, National Changhua University of Education, 1 Jin-De Road, Changhua City, 500 Taiwan; Rothamsted Research, Harpenden, Hertfordshire AL5 2JQ UK; Centre for AgriBioscience, Department of Animal, Plant and Soil Science, School of Life Science, La Trobe University, Bundoora, Australia

**Keywords:** Systemic acquired resistance, Hypersensitive response, Virus movement, RNAi, Post-transcriptional gene silencing, Effector-triggered immunity, Defensive signal transduction

## Abstract

**Background:**

Host RNA-dependent RNA polymerases (RDRs) 1 and 6 contribute to antiviral RNA silencing in plants. RDR6 is constitutively expressed and was previously shown to limit invasion of *Nicotiana benthamiana* meristem tissue by potato virus X and thereby inhibit disease development. RDR1 is inducible by salicylic acid (SA) and several other phytohormones. But although it contributes to basal resistance to tobacco mosaic virus (TMV) it is dispensable for SA-induced resistance in inoculated leaves. The laboratory accession of *N. benthamiana* is a natural *rdr1* mutant and highly susceptible to TMV. However, TMV-induced symptoms are ameliorated in transgenic plants expressing *Medicago truncatula* RDR1.

**Results:**

In *MtRDR1*-transgenic *N. benthamiana* plants the spread of TMV expressing the green fluorescent protein (TMV.GFP) into upper, non-inoculated, leaves was not inhibited. However, in these plants exclusion of TMV.GFP from the apical meristem and adjacent stem tissue was greater than in control plants and this exclusion effect was enhanced by SA. TMV normally kills *N. benthamiana* plants but although *MtRDR1*-transgenic plants initially displayed virus-induced necrosis they subsequently recovered. Recovery from disease was markedly enhanced by SA treatment in *MtRDR1*-transgenic plants whereas in control plants SA delayed but did not prevent systemic necrosis and death. Following SA treatment of *MtRDR1*-transgenic plants, extractable RDR enzyme activity was increased and Western blot analysis of RDR extracts revealed a band cross-reacting with an antibody raised against MtRDR1. Expression of *MtRDR1* in the transgenic *N. benthamiana* plants was driven by a constitutive 35S promoter derived from cauliflower mosaic virus, confirmed to be non-responsive to SA. This suggests that the effects of SA on MtRDR1 are exerted at a post-transcriptional level.

**Conclusions:**

MtRDR1 inhibits severe symptom development by limiting spread of virus into the growing tips of infected plants. Thus, RDR1 may act in a similar fashion to RDR6. MtRDR1 and SA acted additively to further promote recovery from disease symptoms in *MtRDR1*-transgenic plants. Thus it is possible that SA promotes MtRDR1 activity and/or stability through post-transcriptional effects.

**Electronic supplementary material:**

The online version of this article (doi:10.1186/s12870-016-0705-8) contains supplementary material, which is available to authorized users.

## Background

Salicylic acid (SA) is a vital signal molecule involved in maintenance and activation of plant defenses. SA is required for the limitation of pathogen spread during the hypersensitive response (HR), which is a genetically determined resistance mechanism whereby pathogens are restricted to the immediate vicinity of an infection site. Triggering the HR can induce an additional induced resistance mechanism called systemic acquired resistance (SAR) that is also SA-dependent. SAR is effective against a very broad spectrum of pathogens, including viruses, oomycetes, fungi and bacteria [1, 2].

SAR, induced either as the result of the HR or by application of resistance-inducing chemicals, is associated with dramatic changes in the transcriptome [3, 4]. These changes include increased transcription of genes encoding pathogenesis-related (PR) proteins, several of which contribute to defense against fungi, oomycetes and bacteria [5] but not against viruses [6–8]. Indeed, induced resistance to viruses remains poorly understood [9]. SA-induced resistance to viruses is not mediated by any of the known PR proteins and is not dependent on the transcriptional activator ‘Non-Expressor of PR proteins 1’ (NPR1), which is required for *PR* gene induction and effective SA-induced resistance and SAR against non-viral microbial pathogens [10, 11].

One of the mechanisms that could potentially underlie SA-induced resistance to viruses is RNA silencing. The importance of RNA silencing in anti-viral defense may be inferred from the fact that most plant viruses possess counter-defense proteins (viral suppressors of RNA silencing: VSRs) that inhibit the activity or stability of one or more components of the host silencing machinery. The demonstration that the 2b VSR of cucumber mosaic virus (CMV), which inhibits RNA silencing through binding of small RNAs [12–14], also inhibited SA-induced resistance to the replication and local movement of this virus [15], indicated a relationship between SA-induced resistance and silencing. This idea was reinforced by subsequent studies showing that the HC-Pro VSRs of potyviruses can have various effects on SA-mediated resistance to virus spread [16, 17].

An independent line of evidence implicating RNA silencing in SA-induced resistance to viruses was discovered by Xie et al. [18], who found that in tobacco SA increased the accumulation of the transcript encoding RNA-dependent RNA polymerase (RDR) 1 (*NtRDR1*). *Arabidopsis thaliana* was also found to possess an SA-inducible RDR1 gene [19]. RDRs are host enzymes that can initiate or amplify RNA silencing, including antiviral silencing, through synthesis of dsRNA molecules that serve as substrates for Dicer-like (DCL) nucleases [20–22]. The products of DCL-mediated cleavage are short-interfering dsRNAs that, after further processing to ssRNA, direct sequence-specific cleavage or translational arrest of homologous target RNA molecules by Argonaute (AGO) proteins [23, 24]. Plants possess a family of RDR paralogs, with six members occurring in Arabidopsis [25]. RDR1 and RDR6 (which is not affected by SA) have demonstrable roles in limiting virus infection or decreasing virus titre in Arabidopsis and *Nicotiana* species [18, 19, 26–30]. Interestingly, the gene encoding AGO2, one of the AGO proteins with a known antiviral role [31–34], was rendered more sensitive to induction by SA in the presence of a transgene expressing the 2b VSR [4]. This further supports a connection between antiviral silencing and SA-induced resistance.

Although ample evidence suggests a role for silencing in SA-induced resistance, RNA silencing is not absolutely required for successful SA-induced resistance to viruses. It was shown in Arabidopsis that DCLs 2, 3, and 4 are dispensable for SA-induced resistance to TMV and CMV [35]. Thus, it appears that SA induces multiple antiviral systems that include not only RNA silencing but also other mechanisms; for example, the SA-triggered inhibition of virus replication and movement induced via mitochondria-based signaling processes [36, 37].

Meanwhile, the role of RDR1 in virus resistance remains incompletely understood. Transgenic tobacco expressing an anti-sense construct for *NtRDR1* were not compromised in their ability to exhibit SA-induced resistance to tobacco mosaic virus (TMV) and potato virus X (PVX) (reported in [18]). More recently, it was shown that *RDR1* expression is inducible not only by SA but also by a wide range of other phytohormones, including jasmonic acid, and that RDR1 regulates insect resistance in *Nicotiana attenuata* [38–41]. The *Nicotiana benthamiana* accession commonly used for research is a natural *rdr1* mutant that expresses a non-functional form of the enzyme (NbRDR1m), which explains why this plant is hypersusceptible to TMV [42, 43]. Consistent with an important role for RDR1 in virus resistance, TMV-induced disease was ameliorated in transgenic *N. benthamiana* plants constitutively expressing MtRDR1 from *Medicago truncatula* [37, 42]. However, Ying and colleagues [44] presented data showing that transgenic expression of tobacco *NtRDR1* in *N. benthamiana* enhanced plant susceptibility to the potyvirus plum pox virus. This was surprising in the light of previous work showing that down-regulation of *NtRDR1* expression in tobacco plants increased susceptibility to another potyvirus, potato virus Y [29]. To better understand the role of RDR1 in SA-mediated antiviral defense, we explored the effects of SA on virus accumulation and movement and the effects of SA on the gene expression and activity of RDR1 in *N. benthamiana*.

## Results

### Constitutive MtRDR1 expression and SA treatment inhibit spread of TMV in the vicinity of the meristem in *N. benthamiana*

**Fig. 1:**
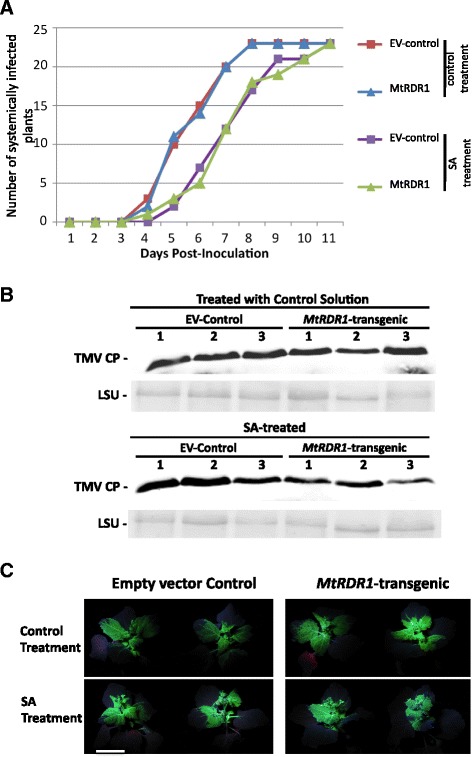
TMV.GFP accumulation in upper, non-inoculated leaves in *N. benthamiana* plants is not prevented by expression of an *MtRDR1*-transgene or by treatment with salicylic acid. Five-week-old *MtRDR1*-transgenic plants (*MtRDR1*), or plants transformed with an ‘empty vector’ transformation vector (EV-control), were pre-treated with a solution of 1 mM SA or a control solution [water amended with 0.05 % (*v/v*) ethanol] prior to inoculation with GFP-tagged TMV. **a** The movement of TMV.GFP from directly-inoculated leaves into non-inoculated leaves was monitored daily using a hand-held UV lamp and the first appearance of GFP fluorescence in non-inoculated leaves recorded. There was no apparent difference in the timing of appearance of TMV.GFP in non-inoculated leaves between the two groups of plants although systemic infection with TMV.GFP was delayed by SA treatment in both types of plant. There were 23 plants in each treatment group. **b** Western blot analysis of TMV.GFP accumulation in systemically-infected leaf tissue of water- (*top*) and SA- (*bottom*) treated plants using anti-TMV coat protein (CP). In each case leaf tissue samples were harvested at 14 days post-inoculation from the uppermost three leaves above the inoculated leaf. Three samples were taken from each treatment group (one sample = one plant). Equal loading of gel lanes with protein is shown by accumulation of ribulose-1, 5-bisphosphate carboxylase/oxygenase large subunit (LSU) revealed by Ponceau S staining of the western blot membrane. **c** The extent of GFP fluorescence in the upper leaves of water-treated TMV.GFP-infected empty vector control (control) and *MtRDR1*-transgenic plants (*MtRDR1*-transgenic) appeared similar when visualised using a hand-held UV lamp and photographed at 14 days post-inoculation. GFP fluorescence was less extensive in SA-treated plants of both groups. The data and photographs above are from one experiment, out of a total of four independent experiments. *Scale bar* = 8 cm

Using TMV engineered to express the green fluorescent protein (TMV.GFP) we investigated the effect of SA on TMV movement in *MtRDR1*-transgenic *N. benthamiana* plants. Five-to-six week old *MtRDR1*-transgenic and empty vector (EV)- control plants (plants from a line transformed with an ‘empty’ transformation vector) were sprayed on the leaves with either 1 mM SA or a control solution once daily for four consecutive days prior to inoculation with TMV.GFP. Plants were monitored daily for the appearance of GFP fluorescence in the upper, non-inoculated leaves. In six independent experiments it was noted that SA treatment consistently resulted in a delay of 1 to 2 days in the first appearance of TMV.GFP in the upper, non-inoculated leaves. However, there was no apparent difference between control and *MtRDR1*-transgenic plants in the timing or patterning of TMV.GFP spread into the upper non-inoculated leaves (Fig. 1a), though *MtRDR1*-transgenic plants were less severely stunted than the control plants (Additional file 1). Western blot analysis of TMV coat protein accumulation in the systemically infected leaves at 14 days post-inoculation (dpi) showed that viral coat protein was present in the upper leaves of both groups of plants (Fig. 1b). When the plants were studied under UV illumination to assess TMV.GFP fluorescence in non-inoculated tissue, there was no marked difference in the timing of first appearance or the apparent extent of GFP fluorescence between control and *MtRDR1*-transgenic *N. benthamiana* plants in the upper leaves (Fig. 1c).

**Fig. 2:**
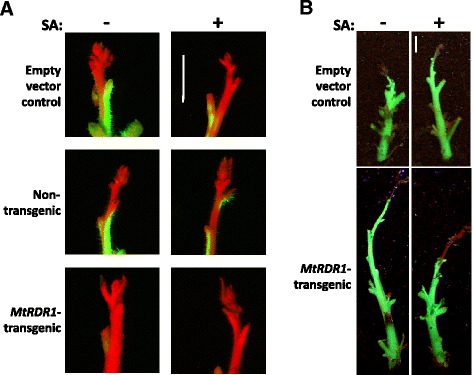
SA treatment enhances RDR1-mediated exclusion of TMV from tissue adjacent to the apical meristem. Empty vector control (plants transformed with an ‘empty’ transformation vector), non-transformed and *MtRDR1*-transgenic plants were treated with 1 mM SA in 0.05 % (*v/v*) ethanol (+) or a control solution of 0.05 % (*v/v*) ethanol (-) prior to inoculation with TMV.GFP. By 14 days post-inoculation the upper leaves of all plants with and without SA treatment showed TMV.GFP fluorescence in the upper leaves. However, when leaves were removed from the stem it was apparent under epifluorescence microscopy (**a**) that spread of TMV.GFP into tissue adjacent to meristems of non-transgenic and control transgenic plants was inhibited by SA treatment and that in *MtRDR1*-transgenic plants, the virus did not enter stem tissue adjacent to the meristem (*scale bar*, 2 mm). (**b**) Stems (with leaves removed) of TMV.GFP infected plants were observed under a UV lamp and photographed with a digital camera, revealing that in *MtRDR1*-transgenic plants the exclusion of TMV.GFP from stem tissue proximal to the meristem was enhanced in SA-treated plants. Photographs in (**a**) and (**b**) are from two independent experiments (*scale bar* = 1 cm)

However, closer examination of the uppermost regions of the stems of SA-treated and untreated control and *MtRDR1*-expressing plants revealed some striking differences in the extent of TMV.GFP spread (Fig. 2). Microscopic observation of fluorescence indicated that SA treatment inhibited the extent of spread of TMV.GFP into the upper stem regions of non-transgenic and EV-control plants (Fig. 2a, Additional file 2). TMV.GFP was excluded entirely from the vicinity of the meristem and much of the upper stems of transgenic plants expressing MtRDR1 (Fig. 2a). Further examination showed that SA treatment enlarged the zone from which TMV.GFP was excluded in *MtRDR1*-transgenic *N. benthamiana* plants (Fig. 2b).

### Recovery of *MtRDR1*-transgenic *N. benthamiana* plants from severe TMV disease was enhanced in SA-treated plants

**Fig. 3:**
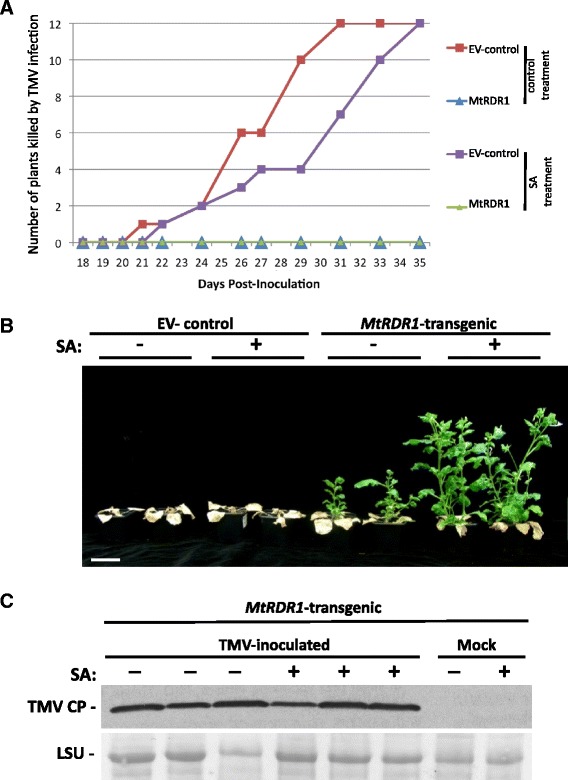
The effects of *MtRDR1* expression and SA treatment on the recovery of *N. benthamiana* plants from TMV-induced plant death. **a** Five-week-old transgenic control (plants transformed with an ‘empty’ transformation vector: EV-control) and *MtRDR1*-transgenic (*MtRDR1*) plants were pre-treated with a solution of 1 mM SA in 0.05 % (*v/v*) ethanol (+SA) or a 0.05 % (*v/v*) ethanol control solution prior to inoculation with TMV U1. Plants were monitored over a 5-week period and the number of plants killed by the virus recorded. With the transgenic control plant group, TMV U1 had killed all plants within 31 days of inoculation. Pre-treatment with 1 mM SA delayed plant death by a few days but did not prevent it. TMV U1-induced plant death was suppressed in all the *MtRDR1*-expressing *N. benthamiana* plants, regardless of whether they were treated with water or with SA. **b** Symptoms at 75 days post-inoculation with TMV U1 in transgenic control and *MtRDR1-*transgenic *N. benthamiana* plants. *MtRDR1*-transgenic plants pre-treated with SA (+) or treated with control solution (-) recovered but SA-treated plant growth was more vigorous (*scale bar* = 8 cm). Data and photographs are from one experiment, out of a total of four independent experiments. **c** Western blot analysis of TMV using anti-TMV coat protein (TMV CP) serum shows that TMV was present in the newly emerged, green leaf tissue in recovering *MtRDR1*-transgenic plants treated with SA (+) or control solution (-). Leaf samples taken at 75 days post-inoculation or mock-inoculation (each lane is loaded with protein extracted from one plant). Equal loading was confirmed by Ponceau S staining of the western blot membrane with the position of the ribulose 1,5-bisphosphate carboxylase/oxygenase large subunit (LSU) indicated

Neither TMV.GFP, nor the TMV.30B vector from which it was derived, induce strong disease symptoms in *N. benthamiana* [45, 46]. However, infection of this host by the U1 strain of TMV causes strong symptoms culminating in systemic necrosis and death of infected plants. Interestingly, TMV-infected *MtRDR1*-expressing *N. benthamiana* plants developed milder symptoms and the plants did not die [37, 42]. Here we found that SA treatment slowed, but did not prevent, TMV-induced death of EV-control transgenic plants and that all plants (SA-treated and untreated) had died by 35 dpi (Fig. 3a). By this time point no *MtRDR1*-transgenic plants had died although they did exhibit TMV U1-induced leaf necrosis, preceded by other symptoms (leaf curling and chlorosis) in many leaves, with the onset of necrosis being slower in SA-treated plants. Necrosis did not become sufficiently extensive to entirely kill *MtRDR1*-transgenic plants. The progress of disease development on *MtRDR1*-transgenic plants was monitored until 75 dpi and it was noted that on all of these plants the spread of necrosis abated and that newly emerging leaves were green and that growth of the plants resumed (Fig. 3b). This apparent recovery from disease was further enhanced on *MtRDR1*-transgenic plants that had been treated with SA prior to inoculation with TMV. SA-treated *MtRDR1*-transgenic plants had grown markedly taller than untreated plants by 75 dpi (Fig. 3b). Western blot analysis using anti-coat protein serum showed that TMV was present in the new leaf tissue produced by the plants (Fig. 3c) indicating that although the *MtRDR1*-expressing transgenic plants recovered from TMV-induced disease and that this recovery was enhanced in SA-treated plants, they did not develop true resistance to the virus.

### SA treatment increased extractable RDR activity in *MtRDR1*-transgenic *N. benthamiana* plants but did not alter *MtRDR1* transcript accumulation

SA had a long-lasting effect on *MtRDR1*-transgenic *N. benthamiana* plants that resulted in an improvement in recovery from TMV-induced disease and resumption of growth (Fig. 3b). This suggested that SA enhances or primes RDR1-mediated defense against TMV. However, *MtRDR1* transgene expression in these plants is under the control of the cauliflower mosaic virus 35S promoter [42] that is, despite containing an as-1 element shared with many SA-responsive plant promoters (for example, that of *PR1a* [47]), not activated by this phytohormone [48].

**Fig. 4:**
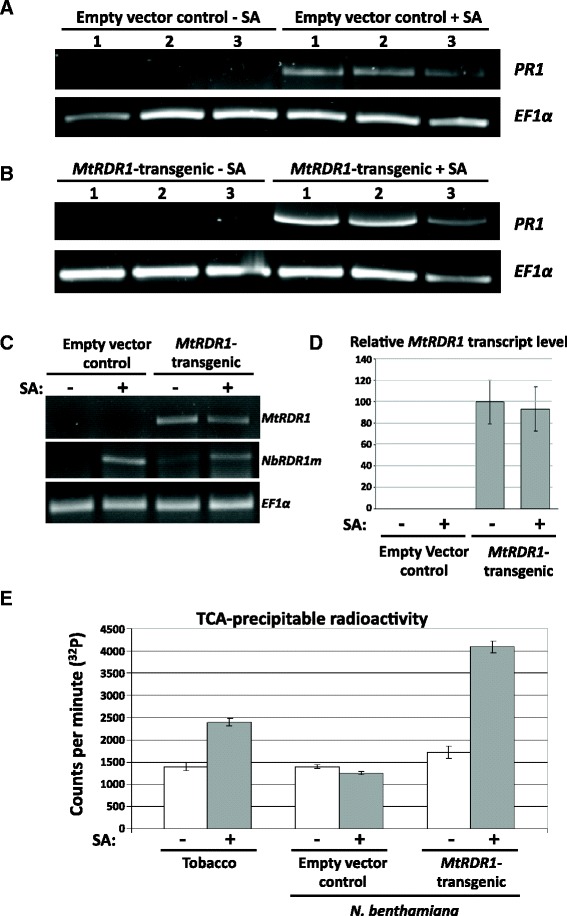
RDR activity, but not *MtRDR1* transcript accumulation, is induced by SA treatment in *MtRDR1*-transgenic *N. benthamiana* plants. Semi-quantitative RT-PCR analysis of *PR1* transcript accumulation in leaves of (**a**) transgenic (empty vector) control and (**b**) *MtRDR1*-transgenic plants infiltrated with a control solution of 0.05 % (*v/v*) ethanol or a solution of 1 mM SA in 0.05 % (*v/v*) ethanol. Infiltrated leaf tissue samples were harvested for RNA extraction at 72 h post-infiltration. *PR1* transcript accumulation levels were determined by RT-PCR after 40 cycles of PCR and compared relative to the accumulation levels of the *elongation factor 1 alpha* (*EF1α*) transcript. Increased *PR1* accumulation confirmed that SA was taken up by the tissues and was effective in inducing transcriptional changes. **c** Semi-quantitative RT-PCR analysis showed that there was little difference in *MtRDR1* transcript accumulation in *MtRDR1*-transgenic plants infiltrated with control solution or 1 mM SA. *NbRDR1m* transcript accumulation was up-regulated in both transgenic control and *MtRDR1*-transgenic *N. benthamiana* plants after SA treatment, although the NbRDR1m protein itself is non-functional. *MtRDR1* and *NbRDR1m* transcript accumulation levels after 27 and 35 cycles, respectively, were compared relative to the accumulation levels of *EF1α*. Infiltrated tissue samples were harvested at 72 h post-infiltration. **d** RT-qPCR analysis of *MtRDR1* transcript levels in leaves of empty vector control and *MtRDR1*-transgenic plants infiltrated with water control or 1 mM SA solution. *MtRDR1* was not detected in empty vector control plants. Mean values for relative *MtRDR1* levels (based on duplicate technical replicate values; 100 = mean value for the transcript level in untreated *MtRDR1* plants) obtained from three plants (one plant = one independent sample) have been given for each treatment group. *Error bars* represent standard errors of the mean for the three samples. Relative transcript levels of *MtRDR1* were calculated using the 2^-ΔΔC(t)^ method [59] using *EF1α* as an internal reference. **e** Enhancement of RDR activity by SA in *MtRDR1*-transgenic plants. Leaves from tobacco (*N. tabacum*, included as a positive control) and transgenic empty vector control and *MtRDR1*-transgenic *N. benthamiana* plants were infiltrated with water (-) containing 0.05 % (*v/v*) ethanol or 2.5 mM SA in 0.05 % (*v/v*) ethanol (+) and harvested after 48 h for preparation of RDR1-enriched extracts. The proxy for RDR1 activity was the incorporation of α-[^32^P] CTP into nascent RNA analysed by liquid scintillation counting of radioactivity incorporated (counts per minute) into trichloroacetic acid-precipitable material. *Error bars* are standard errors for the mean for three technical replicates (RDR assays) per sample

To confirm that the 35S promoter used in the construction of the *MtRDR1*-transgenic plants was behaving as expected, i.e. that it was not responding to SA, we extracted total RNA from leaves of control-transgenic and *MtRDR1*-transgenic *N. benthamiana* plants 72 h after they were infiltrated with either 1 mM SA or a control solution. Semi-quantitative reverse transcription-polymerase chain reaction (RT-PCR) assays showed that SA, but not the control solution, induced expression of the *PR1a* gene, indicating that this SA concentration was effective for the induction of SA-responsive gene expression (Fig. 4a, b). RT-PCR also indicated that whereas the transcript encoding the native, non-functional RDR1 of *N. benthamiana* (NbRDR1m) was induced by SA, the transgene-encoded *MtRDR1* transcript remained at a similar level in *MtRDR1*-transgenic plants in the presence or absence of SA treatment (Fig. 4c). Analysis by RT-quantitative PCR (RT-qPCR) confirmed that steady state levels of *MtRDR1* transcript were not affected by SA treatment in the *MtRDR1-*transgenic *N. benthamiana* plants (Fig. 4d, Additional file 3). Thus, the 35S promoter used in the construction of the *MtRDR1*-transgenic plants is unaffected by SA, meaning that the observed enhancement of RDR1-mediated antiviral action by SA (Figs. 2 and 3b) must be regulated at a post-transcriptional level.

In order to determine if SA treatment affected RDR activity levels, extracts enriched in RDR activity [18] were prepared from non-infected control transgenic and *MtRDR1*-transgenic *N. benthamiana* leaves infiltrated with SA or control solution 48 h prior to tissue harvesting (Fig. 4e). *N. tabacum* (tobacco) possesses the SA-inducible gene *NtRDR1*, which encodes a functional RDR1 [18], and so samples from non-transgenic *N. tabacum* were included in the assays as positive controls for SA-inducible RDR activity. Control-treated EV *N. benthamiana* plants contained active RDR not attributable to RDR1 and as expected, *MtRDR1*-transgenic *N. benthamiana* plants contained a higher level of extractable RDR activity than that detected in the EV control plants (Fig. 4e). Although treatment with SA did not increase the amount of active RDR extractable from EV-control plants (consistent with the known absence of SA-inducible RDR activity in *N. benthamiana* [42]), SA treatment did increase RDR activity in *MtRDR1-*transgenic plants. This effect was only observed when SA was applied to intact plant tissue, and not when RDR extracts from untreated *N. tabacum* or *MtRDR1*-expressing *N. benthamiana* plants were incubated in vitro with SA or with a biologically inactive isomer of SA (Additional file 4).

### MtRDR1 protein is detectable in RDR extracts from SA-treated *MtRDR1*-transgenic *N. benthamiana* plants

**Fig. 5:**
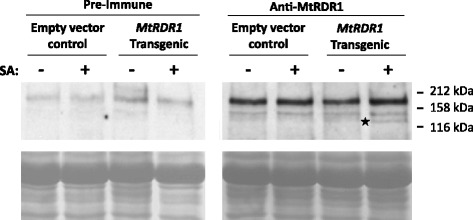
Western blot analysis for MtRDR1 in RDR1-enriched protein extracts. Leaves of empty vector control transgenic or *MtRDR1*-transgenic *N. benthamiana* plants were infiltrated with a control solution of water containing 0.05 % (*v/v*) ethanol (-) or 2.5 mM SA (+). RDR1-enriched protein extracts were prepared 48 h later and subjected to western blot analysis with either preimmune rabbit serum (*left panel*) or polyclonal rabbit anti-MtRDR1 serum (*right panel*). The positions of protein molecular mass markers are indicated (kDa). A protein (indicated with a *star*) corresponding to the predicted size of 131 kDa for MtRDR1 was specifically detected in SA-treated *MtRDR1*-transgenic plants with the anti-MtRDR1 serum but not with the preimmune serum. Equal loading of gel lanes with protein, based on the Bradford [59] assay was confirmed by Ponceau S staining of the western blot membrane (*lower panels*)

Since the in vitro RDR activity of extracts from *MtRDR1*-transgenic plants was increased approximately 2.5 fold when the plants were pre-treated with SA, we investigated whether there was any change in the accumulation of MtRDR1 protein in these extracts. A polyclonal anti-MtRDR1 rabbit serum was prepared using a maltose binding protein (MBP) MtRDR1 fusion protein made in *Escherichia coli* (Additional file 5). This was used for Western blot analysis of RDR-enriched extracts (Fig. 5). As the anti-MtRDR1 serum detected a number of background plant protein bands, western blot analysis with preimmune serum was also carried out (Fig. 5). The only polypeptide specifically detected by the anti-MtRDR1 serum was of *c*.131 kDa, the mass predicted for MtRDR1 (Additional file 6), and this was present only in RDR-enriched extracts from SA-treated, *MtRDR1*-transgenic plants (Fig. 5). Ubiquitination and multiple phosphorylation sites are predicted to occur in the MtRDR1 protein sequence (Additional file 7). This raises the possibility that SA treatment stabilizes the protein (through phosphorylation or inhibition of ubiquitin-mediated breakdown) or, alternatively, that SA triggers recruitment of MtRDR1 into an active complex.

## Discussion

We studied the effects of RDR1 on the long-distance movement and spread of TMV. Expression of MtRDR1 in *N. benthamiana* did not prevent TMV.GFP movement into non-inoculated leaf tissue or enhance the inhibitory effect of SA on virus movement into these tissues. However, MtRDR1 expression inhibited the extent of spread into the region proximal to the apical meristem and this movement restriction became more pronounced following treatment of *MtRDR1*-transgenic plants with SA. Schwach and colleagues [28] showed that knock-down of *NbRDR6* gene expression in the same host (*RDR6i*-transgenic *N. benthamiana*) allowed entry of PVX into the meristem and that this resulted in an exacerbation of PVX-induced disease symptoms throughout the plant. In line with previous work [49] we found that in *N. benthamiana* plants, TMV.GFP enters tissue adjacent to the meristem, although it does not appear to enter the meristem itself. In *MtRDR1*-transgenic plants, however, the ability of TMV.GFP to approach the meristem is drastically curtailed. Furthermore, in the *MtRDR1*-transgenic plants the normally lethal effect of TMVU1 is ameliorated and recovery from disease occurs. Our results with constitutive expression of *MtRDR1* complement and extend the results and conclusions of Schwach and colleagues [28]; indicating that RDR1, as well as RDR6, can function to inhibit virus entry to tissue adjacent to the meristem. Both studies point to a relationship between the effectiveness of RNA silencing-mediated exclusion of virus from meristematic and adjacent tissues with the severity of virus-induced disease symptoms. This suggests that, with respect to the elaboration of disease symptoms, that there is a critical developmental stage for plant cells and tissues, which corresponds physically to a certain point along the sub-apical zone. If viral entry into these tissues is delayed, for example by host defense mechanisms, until after this critical developmental stage has been completed, plants will to some extent be able to recover from virus infection.

It has been postulated that the hyper-susceptibility of the laboratory accession of *N. benthamiana* to a number of tobamoviruses including TMV can be attributed to the absence of an active RDR1 in this species [42]. Ying and colleagues [43] proposed an alternative hypothesis, suggesting that RDR1 can enhance virus spread and accumulation and that the loss of RDR1 functionality in *N. benthamiana* may be due to selective pressure to maintain high levels of RDR6-dependent antiviral defense. This hypothesis was formulated mainly to explain results after challenge with non-tobamoviruses, as challenge with TMV did not alter symptoms and at best only modestly decreased virus accumulation in systemically-infected tissue of *N. benthamiana* plants expressing NtRDR1 [43]. We have seen no increase in susceptibility to TMV in our study of *N. benthamiana* plants expressing the transgene derived from the RDR1 of *M. truncatula*. Furthermore, wild accessions of *N. benthamiana* that contain a non-truncated, functional NbRDR1 are naturally protected against severe symptoms from tobamovirus infection and are not more susceptible to poty- or cucumovirus infection than *N. benthamiana* carrying *NbRDR1m* (43) indicating that RDR1 does indeed have a particular role in ameliorating tobamovirus infection in *N. benthamiana*.

A difference in the effects of RDR1 and RDR6 on virus spread is apparent in the pattern of spread of PVX.GFP into upper uninoculated leaves previously observed in *RDR6i* plants [28] and in the pattern of systemic TMV.GFP movement in *MtRDR1*-transgenic plants in the present study (Fig. 1). Specifically, knock-down of *NbRDR6* accelerated entry of virus into well-developed upper leaves, whereas constitutive expression of MtRDR1 did not slow the appearance of TMV.GFP in these leaves. These differences suggest that the roles of RDRs 1 and 6 do not overlap completely and that each may be effective in protection against different viruses with differing infection and movement strategies, as suggested previously [42].

These results suggest that RDR1 may play a role similar to RDR6 in protecting the meristem from viral invasion. The strong *AtRDR1* promoter activity in Arabidopsis phloem tissue observed by Xu and colleagues [41] may also help restrict the spread of virus into the vasculature and subsequently the meristem, although curiously the *AtRDR1 promoter:β-glucuronidase* reporter transgene was found to be poorly active in younger tissues. However, since *RDR1* gene expression is induced by chemical signals associated with SAR (SA, nitric oxide and hydrogen peroxide) [39, 40] as well as by many other phytohormones (jasmonic acid, abscisic acid, auxin, ethylene) and wounding [18, 39, 41], it may be that the multiplicity of factors affecting RDR1 expression, and its therefore complex regulation, allows plants to reinforce meristem protection against invasion in times of stress or perhaps control the accessibility of this tissue to endogenous factors during development.

Previous studies showed that plants expressing a functional RDR1 are less susceptible to TMV-induced disease, yet RDR1 does not seem to be required for successful SA-induced resistance to viruses in directly inoculated leaves [18, 19, 37]. Interestingly, we found here not only that expression of *MtRDR1* enabled *N. benthamiana* to recover from severe disease induced by TMV strain U1, but also that this recovery was enhanced by SA treatment. Whilst this could have been due to the activity of one or more of the RDR1-independent SA-inducible antiviral defense systems that are known to exist [37], SA treatment of control-transgenic *N. benthamiana* plants only delayed TMV-induced plant death by a few days suggesting that the protective activity of other SA-induced antiviral defense systems was insufficient in this case to account for the marked enhancement of recovery after SA treatment in the *MtRDR1-*transgenic *N. benthamiana* plants.

Although SA and MtRDR1 worked synergistically in protection against severe TMV-induced disease, it is not possible to say with certainty whether or not these factors were working together or separately. However, the hypothesis that SA enhanced RDR1 activity was favored by the observation that *MtRDR1*-transgenic plants contained more extractable RDR activity following SA treatment, despite there being no concomitant SA-induced increase in *MtRDR1* transgene expression. There was no direct effect of SA on in vitro RDR activity in plant extracts (Additional file 4), which indicates that SA indirectly influences RDR activity *in planta*. Detection of a band cross-reacting with an antibody raised against MtRDR1 in RDR1-enriched preparations from SA-treated but not untreated *MtRDR1*-transgenic plants was consistent with the detection of greater RDR1 activity. It also suggested that RDR1 can be regulated by SA through post-translational process(es) either resulting in a more stable MtRDR1 or involving recruitment of MtRDR1 protein to a complex. An *in silico* analysis of the MtRDR1 protein sequence revealed a number of candidate ubiquitination and phosphorylation sites (Additional file 7). Post-translational protein modification plays an important role in host immunity [50, 51], for example: ubiquitin-dependent proteolysis negatively regulates defense responses in tobacco [52]; NPR3 and 4 are E3 ligase adapters that control NPR1 activity by proteolysis in an SA-dependent manner in Arabidopsis [53, 54]; and SUMO proteases negatively regulate SA biosynthesis [55]. It may therefore be that SA induces, in some manner, stabilization of the MtRDR1 protein against small modifier protein-mediated degradation and/or activation/deactivation of the protein by phosphorylation. The idea of assembly of a complex, which includes RDR1 and forms in response to SA, is made more plausible by recent findings that several RNA silencing factors, including RDR6, form complexes with each other and with other cellular components such as membranes [56, 57]. Another possibility is that SA enhances translation of the *MtRDR1* transcript leading to increased synthesis of MtRDR1 protein but it is difficult to envisage a likely mechanism for this.

## Conclusions

In conclusion, our results support an important role for the phytohormone-inducible factor RDR1 in resistance by limiting viral access to the region adjacent to the meristem and thereby ameliorating the severity of virus-induced disease. Its ability to inhibit access to the meristematic region correlates with amelioration of TMV-induced disease symptoms and is enhanced by SA. Our data suggests that SA may enhance RDR1 activity at a post-transcriptional level in addition to the previously documented effects of this phytohormone on transcription of the *RDR1* gene.

## Methods

### Plant growth conditions

Seeds of the laboratory accession of *Nicotiana benthamiana* Domin. [42] non-transgenic, control transgenic (Line V19-1) and *MtRDR1*-transgenic (Line R15-1) plant lines [43] and of tobacco (*N. tabacum* L. cv. Xanthi) were germinated in soil and plants cultivated in a controlled environment room (Conviron Ltd., Winnipeg, Manitoba, Canada) with a 16 h photoperiod (200 μE.m^2^.s^−1^ of photosynthetically active radiation) at 22 °C and 60 % relative humidity.

### Virus strains, chemical treatments and detection of infection

The viral strains used in this study were the common (U1) strain of TMV [58], and TMV.GFP (TMV30B.GFP constructed by Shivprasad et al. [45]). Capped TMV.GFP infectious RNA was re-generated by in vitro transcription as previously described and inoculated onto 3- to 5-week old *N. benthamiana* plants to prepare aliquots of infectious sap as described by Murphy et al. [46]. For whole-plant treatments with SA, 5-to-6 week old *N. benthamiana* plants were sprayed for four consecutive days with either a control solution [0.05 % (*w/v*) ethanol] or 1 mM SA dissolved in 0.05 % (*w/v*) ethanol before mechanical inoculation with TMV U1 or TMV.GFP on one or two lower leaves [37]. Treatment of leaf tissue by infiltration was carried out as described previously using 2.5 mM SA dissolved in 0.05 % (*w/v*) ethanol [37]. Western blot detection of TMV using anti-TMV coat protein serum was carried out using a previously described method [46]. Observations of GFP fluorescence utilized either a hand-held UV lamp and Nikon Cool-Pix digital camera or epifluorescence microscopy using a Nikon Optiphot 2 (Nikon Ltd., Kingston-upon-Thames, UK) [59].

### RNA extraction, cDNA synthesis and PCR

Total RNA was prepared [60] and treated with DNase I using the TURBO DNase-*free* kit (Ambion, Austin, TX). First-strand cDNA synthesis was carried out with 1 μg treated RNA using Superscript III (Invitrogen, Paisley, UK) with random pentadecamer primers according to the manufacturer’s instructions. Primers for semi-quantitative or quantitative PCR are listed as a table in Additional file 8. For semi-quantitative RT-PCR analysis of plant gene expression cDNA was amplified with RedTaq DNA polymerase (Sigma-Aldrich, Poole, UK) using the following conditions: 1 cycle of 94 °C for 2 min; 27 to 40 cycles of 94 °C for 15 s, 55 °C for 30 s, 72 °C for 40 s; and 1 cycle of 72 °C for 5 min. PCR products were analysed by electrophoresis on 1.5 % agarose gels. For RT-qPCR analysis of *MtRDR1* transcript levels, the cDNA template was diluted 1:5 and qPCR performed using SYBR Green JumpStart Taq ReadyMix (Sigma-Aldrich, St Louis, MO) according to the manufacturer’s instructions. Reactions were conducted in duplicate (40 cycles: 94 °C for 15 s, 57 °C for 30 s, 72 °C for 40 s) on a Chromo4 PCR system (Bio-Rad, Hemel-Hempsted, UK) and analysed using the LinRegPCR program [61]. Relative transcript levels of *MtRDR1* were calculated using the 2^-ΔΔC(t)^ method [62] using *NbEF1α* as an internal reference (previously authenticated for stability in the presence of SA [63]).

### Isolation and assay of RDR activity

The isolation of RDR1-enriched extracts and assays for RDR activity were carried out as described by Xie and colleagues [18]. Leaf tissue (2 g fresh weight) from 21 days-old *N. tabacum* or *N. benthamiana* plants was harvested 48 h post-infiltration with 1 mM SA in 0.05 % (*w/v*) ethanol or control solution [0.05 % (*w/v*) ethanol]. Tissue was homogenised in 4 ml of buffer A (50 mM Tris-acetate pH 7.4, 10 mM potassium acetate, 1 mM EDTA, 10 mM 2-mercaptoethanol and 0.5 mM phenylmethysulfonylfluoride), centrifuged at 1000 × *g* for 12 min and centrifuged again at 14,000 × *g* for 10 min after adding glycerol to a final proportion of 20 % (*v/v*). An equal volume of 4 M (NH_4_)_2_SO_4_ was added to each supernatant and the proteins precipitated over 2 h with gentle agitation (50 rpm) on an orbital shaker. Precipitated protein was collected by centrifugation at 10,000 × g for 20 min. Pellets were washed briefly with 1 ml buffer B [25 mM Tris-acetate pH 8.2, 1 mM EDTA, 20 % (*v/v*) glycerol and 3 mM 2-mercaptoethanol] twice. The RDR1-enriched protein extracts were re-suspended in 300–500 μl buffer B and dialysed overnight against buffer B. All preparation steps were carried out at 4 °C. Protein concentrations were assayed using the method of Bradford [64] with a Bio-Rad protein quantification kit.

In vitro RDR assays were performed in a 50 μl final volume containing 25 μg of RDR1-enriched protein preparation with 50 mM Tris–HCl (pH 8.0), 10 mM MgCl_2_, 1 mM DTT, 1 mM each of ATP, GTP, UTP, and 2 μM CTP supplemented with α-[^32^P]CTP (Perkin-Elmer, Little Chalfont, UK) and 4 μg total RNA from TMV-infected *N. tabacum* leaves [18]. Reactions were initiated by adding the RDR1-enriched protein preparation and incubated for 2 h at 30 °C. Reactions were terminated by incubation at 95 °C for 5 min and placed on ice. Incorporation of α-[^32^P]CTP into RDR reaction products was quantified by scintillation counting of radioactivity incorporated into trichloroacetic acid-precipitable material [65] using OptiPhase Hisafe 3 scintillation cocktail (Perkin-Elmer) and a 2000 Tri-CARB liquid scintillation analyser (Packard, Illinois, USA). RDR assays were carried out using at least three biological replicates.

### Expression of recombinant MtRDR1 in *Escherichia coli* and production of rabbit polyclonal anti-MtRDR1

The plasmid pMAL-MtRDR1 was obtained by cloning the MtRDR1 in pMAL-c2E (New England Biolabs, MA, USA) in frame to the carboxyl terminus of the MBP coding sequence using the KpnI and HindIII restriction enzyme sites. Cells of *E. coli* TB1 strain (NEB) were co-transformed with pMAL-MtRDR1 and the CodonPlus plasmid (Stratagene). CodonPlus plasmids contain genes for tRNAs that recognize the arginine codons AGA and AGG, the isoleucine codon AUA, and the leucine codon CUA, respectively, which improves the availability of tRNAs that most frequently restrict translation of AT-rich eukaryotic genes in *E. coli*. The recombinant protein expressed by the pMAL-RDR1 clone was induced and purified by amylose resin column chromatography  as described in the manufacturer’s instructions (NEB) (Additional file 5). The amylose column fractions were further purified on a HiLoad Superdex200 column attached to an AKTA purification system (GE Healthcare Life Sciences, USA). Purified fractions were run on a SDS-PAGE gel and stained with Coomassie Brilliant Blue R-250 (Additional file 5). Putative MBP-MtRDR1 fusion protein was extracted from the SDS page gel and verified by MALDI-TOF MS [66] (data not shown). Gel slices containing 1 mg of MtRDR1 protein were sent to Covance Inc. (Denver, PA, USA) for antibody production, where two rabbits (OK127 and OK129) were injected with 125 μg each of MtRDR1 protein at 14, 35, 49 and 70 days following a sampling for pre-immune serum. Test bleeds were collected on the 45th day and production bleeds were collected on the 59th and 77th days. The test bleed and production bleeds were purified by using NAb Protein A/G Spin Columns (Pierce, USA). Antibody obtained from rabbit OK129 exhibited better activity in the preliminary experiments (data not shown). Thus, anti-MtRDR1 and the corresponding pre-immune serum from rabbit OK129 were used in the current study.

### Western blotting for MtRDR1

RDR1-enriched protein extracts were prepared as above and equal amounts of protein analyzed by SDS-PAGE on 15 % (*w/v*) acrylamide gels [67] before electrophoretic transfer to nitrocellulose [68]. Equal loading of protein samples was confirmed using Ponceau S staining. Western blot analysis of MtRDR1 accumulation was carried out using a rabbit polyclonal anti-MtRDR1 serum (1:10,000 dilution) or control preimmune rabbit serum (1:10,000 dilution) as primary antibodies followed by anti-rabbit IgG conjugated to horseradish peroxidase (1:15,000 dilution) secondary antibody. Bound antibody was detected using the Western Lightning Chemiluminescence Reagent Plus (PerkinElmer) and exposure to Konica Minolta AX film (Konica Minolta Medical and Graphic, Japan).

### *In silico* predictions of phosphorylation and ubiquitination sites in the MtRDR1 sequence

*In silico* translation of the *MtRDR1* cDNA sequence was carried out using the ExPASy Translate tool (http://web.expasy.org/translate/) from the Swiss Institute of Bioinformatics ExPASy Bioinformatics Resource Portal. Potential phosphorylation sites in the MtRDR1 protein sequence were predicted using NetPhos 2.0 software (www.cbs.dtu.dk/services/NetPhos/) [69]. NetPhos 2.0 generates a score in the range 0.000 to 1.000 to indicate the likelihood of a serine, threonine or tyrosine being a phosphorylation site, with residues with a score >0.500 being assigned as potential phosphorylation sites. UbPred software (http://omictools.com/ubiquitination-sites-category) was used to predict potential ubiquitination sites [70]. Residues with ubiquitination scores >0.62 are assigned as potential ubiquitination sites, with scores in the range 0.62–0.69 being low confidence predictions and those in the range 0.69–0.84 being high confidence predictions.

### Availability of supporting data

The data sets supporting the results of this article are included within the article and its additional files.

## Additional files

Additional file 1:
**Symptom severity in TMV-GFP infected** ***Nicotiana tabacum*** **.** (PDF 10005 kb) Additional file 2:
**Extent of TMV.GFP spread into** ***Nicotiana tabacum*** **stem tissue adjacent to the shoot apical meristem.** (PDF 2233 kb) Additional file 3:
**Relative MtRDR1 transcript level in MtRDR1 transgenic** ***Nicotiana tabacum*** **after SA treatment.** (PDF 48 kb) Additional file 4:
**RDR activity in extracts from** ***Nicotiana tabacum*** **and** ***Nicotiana benthamiana*** **plants is not increased by in vitro treatment with SA.** (PDF 484 kb) Additional file 5:
**Expression of a MBP-MtRDR1 fusion protein in** ***E. coli*** **and its purification by amylose affinity chromatography and gel filtration.** (PDF 635 kb) Additional file 6:
**Calibration curve to determine the molecular weight (log**_**10**_ **MW) of protein detected by anti-MtRDR1 serum on SDS-PAGE gel.** (PDF 72 kb) Additional file 7:
**Predicted ubiquitination and phosphorylation sites in the MtRDR1 amino acid sequence.** (PDF 153 kb) Additional file 8:
**Table of primers used for semi-quantitative and quantitative PCR.** (PDF 76 kb)

## Abbreviations

AGO: Argonaute
CMV: cucumber mosaic virus
DCL: Dicer-like nucleases
EV: empty vector
GFP: green fluorescent protein
HR: hypersensitive response
MBP: maltose binding protein
NPR1: Non-Expressor of PR proteins 1
PR: pathogenesis-related proteins
PVX: potato virus X
RDR: RNA-dependent RNA polymerases
SA: Salicylic acid
SAR: systemic acquired resistance
TMV: tobacco mosaic virus
VSR: viral suppressors of RNA silencing
