# Supplementary material for: Salicylic acid treatment and expression of an RNA-dependent RNA polymerase 1 transgene inhibit lethal symptoms and meristem invasion during tobacco mosaic virus infection in Nicotiana benthamiana
Source: BMC Plant Biol. 2016 Jan 13;16:15. doi: 10.1186/s12870-016-0705-8 (PMC4710973; doi:10.1186/s12870-016-0705-8)
Supplement: Additional file 1: — Symptom severity in TMV-GFP infected Nicotiana tabacum . (PDF 10005 kb) [file 12870_2016_705_MOESM1_ESM.pdf]

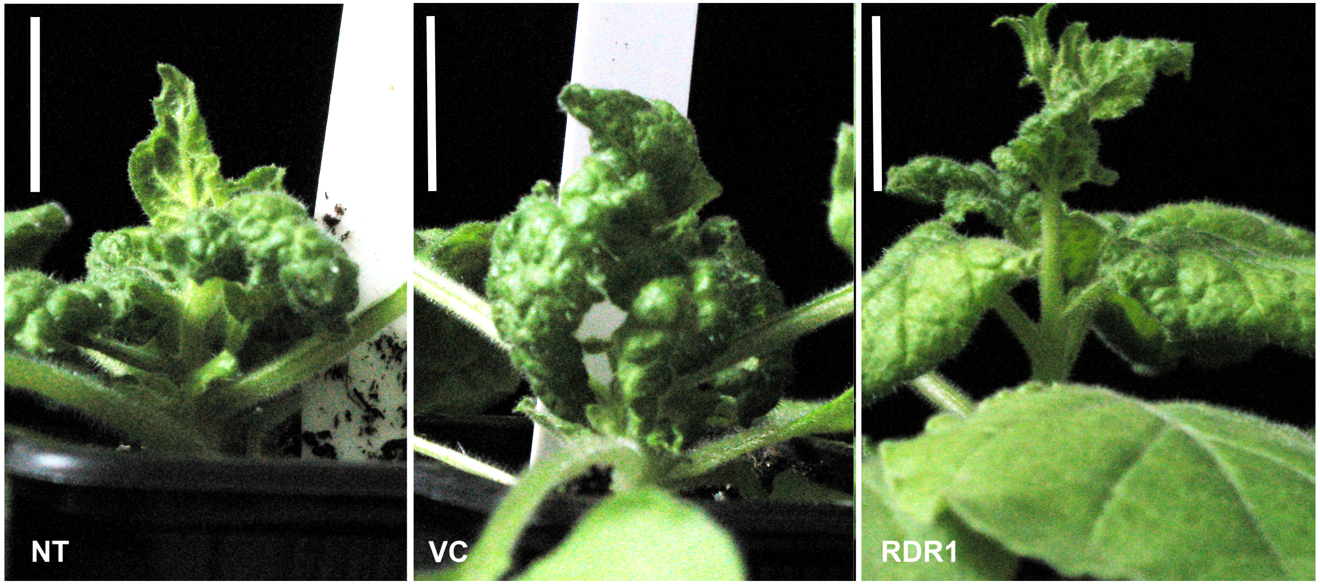

Additional File 1

Comparison of symptom severity in *N. benthamiana* 15 days post inoculation with TMV.GFP, showing that transgenic *N. benthamiana* expressing MtRDR1 is less severely stunted compared to the non-transgenic and empty-vector control plants.
NT= non transgenic plant.
VC= vector control transgenic plant.
RDR1= Transgenic *N. benthamiana* expressing MtRDR1.
Scale bar = 1.5 cm.
