# Supplementary material for: Salicylic acid treatment and expression of an RNA-dependent RNA polymerase 1 transgene inhibit lethal symptoms and meristem invasion during tobacco mosaic virus infection in Nicotiana benthamiana
Source: BMC Plant Biol. 2016 Jan 13;16:15. doi: 10.1186/s12870-016-0705-8 (PMC4710973; doi:10.1186/s12870-016-0705-8)
Supplement: Additional file 2: — Extent of TMV.GFP spread into Nicotiana tabacum stem tissue adjacent to the shoot apical meristem. (PDF 2233 kb) [file 12870_2016_705_MOESM2_ESM.pdf]

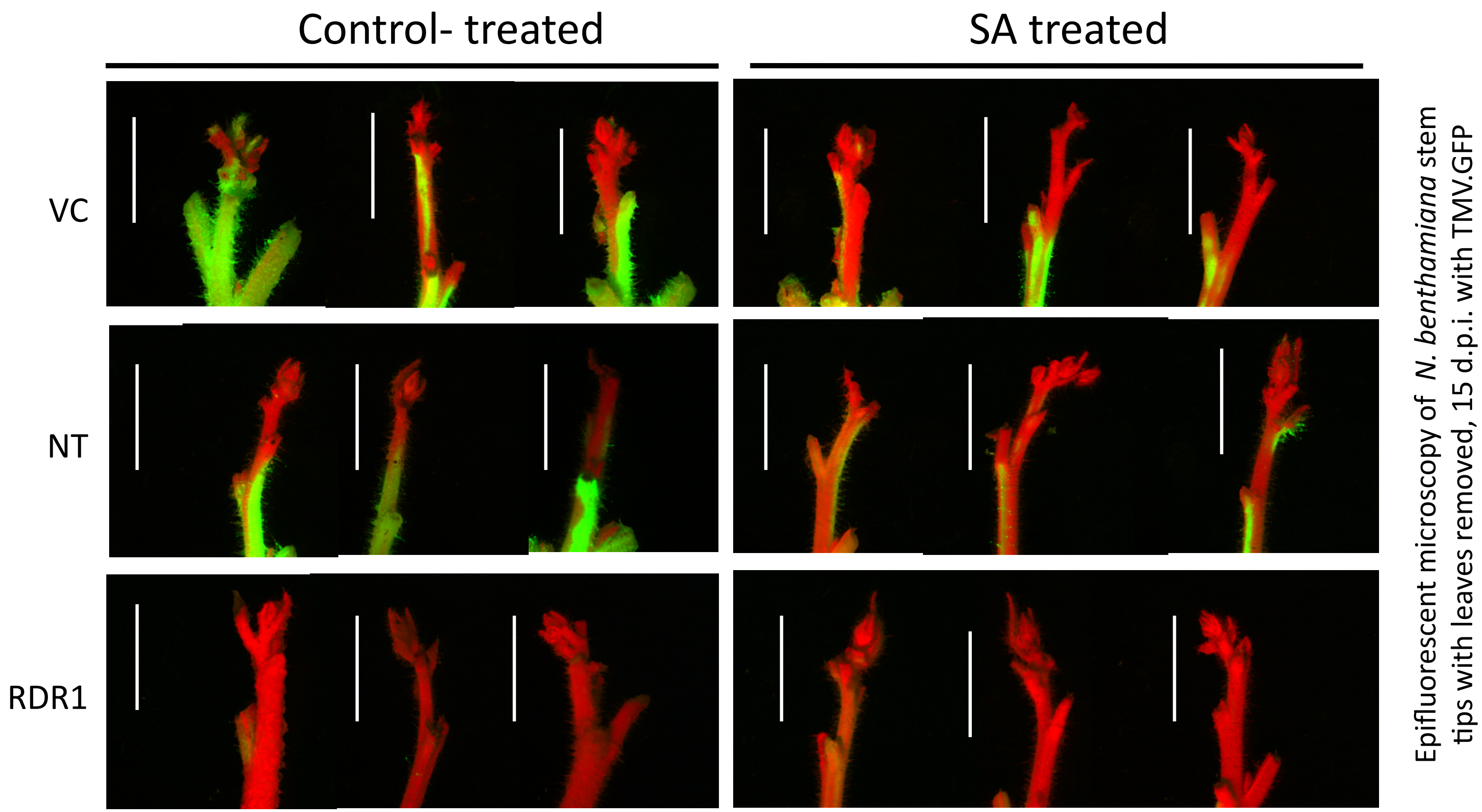


Additional File 2

Transgenic *N. benthamiana* plants expressing *MtRDR1* prevents infection of the stem tissue adjacent to the meristem by TMV.GFP.
scale bar = 3mm
