# Supplementary material for: Salicylic acid treatment and expression of an RNA-dependent RNA polymerase 1 transgene inhibit lethal symptoms and meristem invasion during tobacco mosaic virus infection in Nicotiana benthamiana
Source: BMC Plant Biol. 2016 Jan 13;16:15. doi: 10.1186/s12870-016-0705-8 (PMC4710973; doi:10.1186/s12870-016-0705-8)
Supplement: Additional file 3: — Relative MtRDR1 transcript level in MtRDR1 transgenic Nicotiana tabacum after SA treatment. (PDF 48 kb) [file 12870_2016_705_MOESM3_ESM.pdf]

Relative *MtRDR1* transcript level in transgenic *MtRDR1* *N. benthamiana* after SA treatment

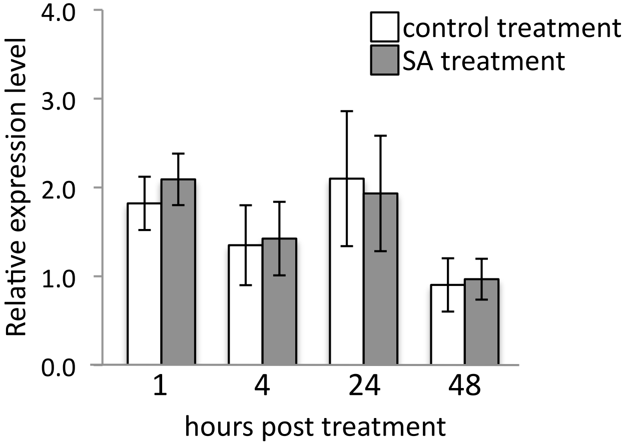

Additional File 3

RT-qPCR analysis of *MtRDR1* transcript levels in leaves of *MtRDR1*-transgenic plants at 1, 4, 24 and 48 hours infiltration with control or 1 mM SA solution compared to transcript levels at the start of the experiment. *MtRDR1* was not detected in vector control plants. Mean values for *MtRDR1* levels were obtained from three plants (one plant = one independent sample). Error bars represent standard errors of the mean for the three samples. Relative transcript levels of *MtRDR1* were calculated using the $2^{-\Delta\Delta C(t)}$ method [59] using *EF1α* as an internal reference. In a one way ANOVA, there was no significant difference in *MtRDR1* transcript levels between the control and SA treatments.
