# Supplementary material for: Salicylic acid treatment and expression of an RNA-dependent RNA polymerase 1 transgene inhibit lethal symptoms and meristem invasion during tobacco mosaic virus infection in Nicotiana benthamiana
Source: BMC Plant Biol. 2016 Jan 13;16:15. doi: 10.1186/s12870-016-0705-8 (PMC4710973; doi:10.1186/s12870-016-0705-8)
Supplement: Additional file 4: — RDR activity in extracts from Nicotiana tabacum and Nicotiana benthamiana plants is not increased by in vitro treatment with SA. (PDF 484 kb) [file 12870_2016_705_MOESM4_ESM.pdf]

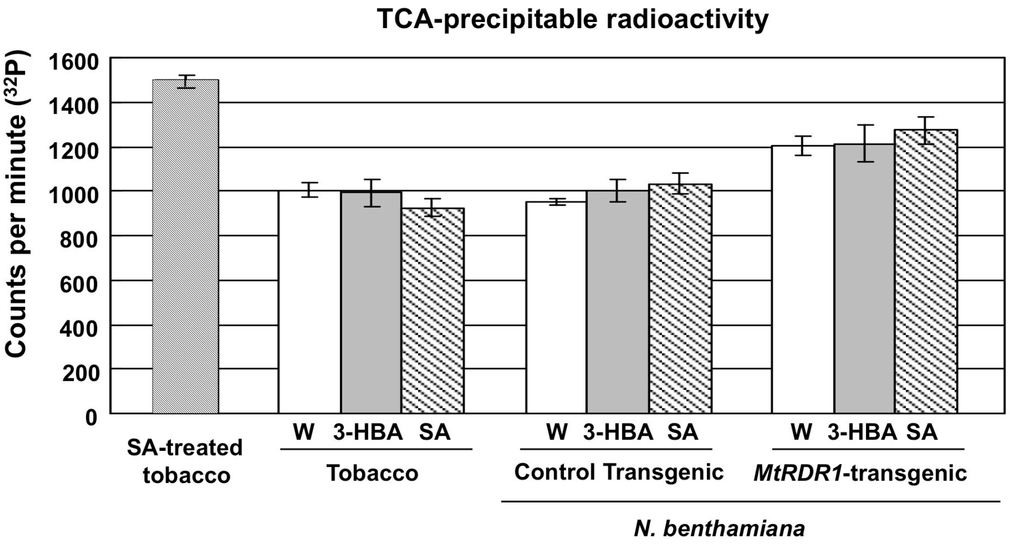

Additional File 4: RDR activity in extracts from tobacco and *Nicotiana benthamiana* plants is not increased by *in vitro* treatment with SA. Leaves of tobacco (*N. tabacum*), control-transgenic and *MtRDR1*-transgenic *N. benthamiana* plants were harvested 48 hours post-infiltration with water and RDR1-enriched protein extracts isolated. SA or 3-hydroxybenzoic acid (3-HBA, a biologically inactive isomer of SA) at final concentrations of 2.5mM, or water (W) amended with 0.05% (v/v) ethanol was added to the plant protein extracts immediately before they were assayed for RDR activity. RDR assays were performed in a reaction containing α-[^{32}P] CTP, NTPs and RNA extracted from TMV-infected tobacco plants. The proxy for RDR activity was the incorporation of α-[^{32}P] CTP into nascent RNA analysed by liquid scintillation counting of radioactivity incorporated (counts per minute) into trichloroacetic acid (TCA)-precipitable material. An NtRDR1-enriched protein extract from SA-infiltrated tobacco leaves was used as a positive control (SA-treated tobacco). Error bars are standard errors for the mean for three technical replicates (RDR assays) per sample.
