# Supplementary material for: Salicylic acid treatment and expression of an RNA-dependent RNA polymerase 1 transgene inhibit lethal symptoms and meristem invasion during tobacco mosaic virus infection in Nicotiana benthamiana
Source: BMC Plant Biol. 2016 Jan 13;16:15. doi: 10.1186/s12870-016-0705-8 (PMC4710973; doi:10.1186/s12870-016-0705-8)
Supplement: Additional file 5: — Expression of a MBP-MtRDR1 fusion protein in E. coli and its purification by amylose affinity chromatography and gel filtration. (PDF 635 kb) [file 12870_2016_705_MOESM5_ESM.pdf]

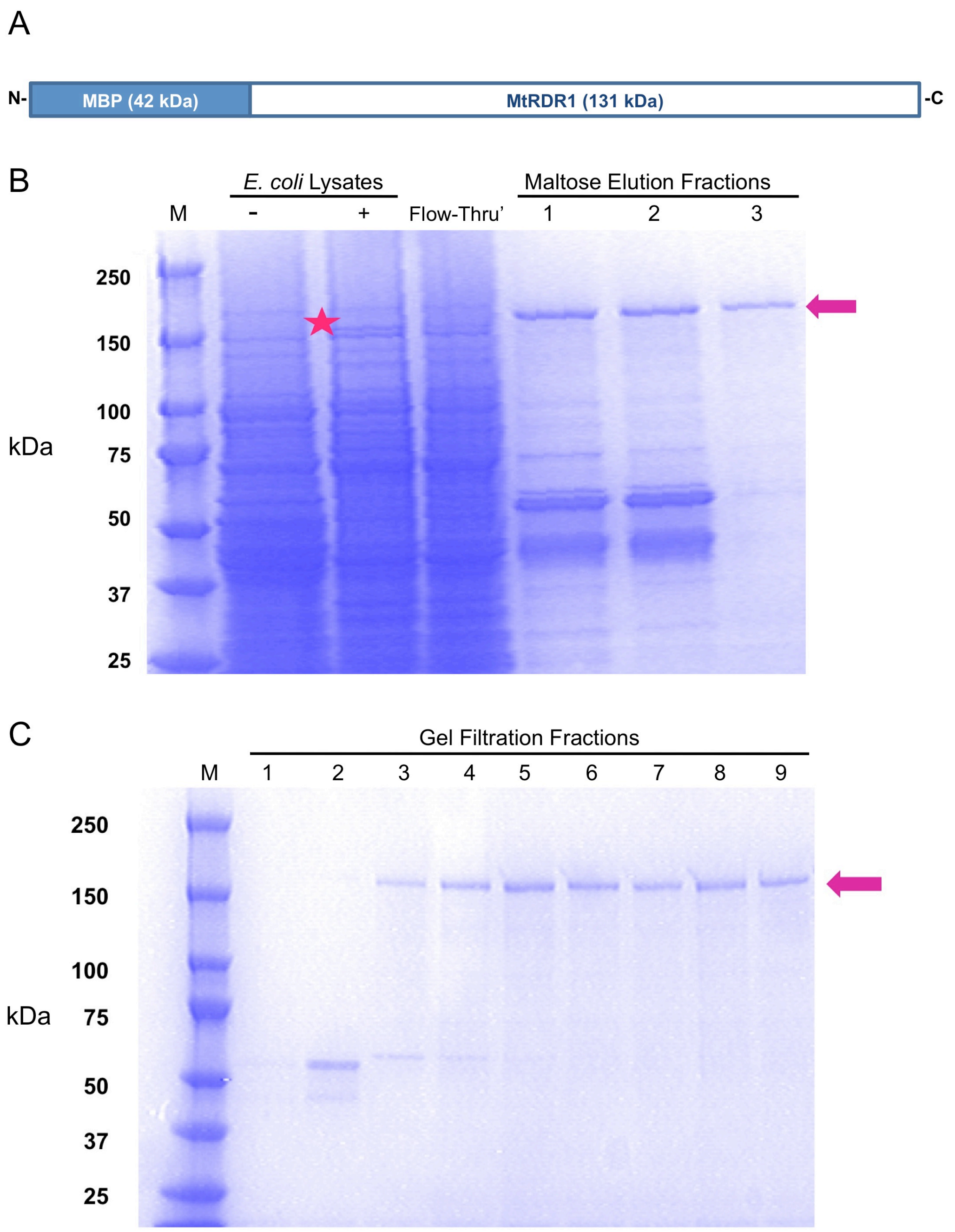


**Additional File 5: Expression of a MBP-MtRDR1 fusion protein in *E. coli* and its purification by amylose affinity chromatography and gel filtration.** (A) Schematic representation of the recombinant MBP-MtRDR1 fusion protein produced in *E. coli* with approximate molecular weight values in parentheses. (B) SDS-polyacrylamide gel electrophoresis (SDS-PAGE) of: cell lysates of *E. coli* transformed with the vector harboring the insert encoding the MBP-MtRDR1 fusion

protein (pMAL-MtRDR1: indicated by +) or the parent vector lacking the insert (pMAL-c2E: indicated by -); lysate proteins from pMAL-MtRDR1-transformed cells that were unbound by an amylose affinity chromatography column (Flow-Thru'), and lanes (5, 6, and 7) loaded with proteins eluted by washing with maltose solution. M indicates a lane loaded with protein standard (molecular weight values indicated in kDa). Star and arrow indicate position of polypeptide band corresponding to the recombinant protein (c. 173 kDa). Polypeptide bands were visualized by staining with Coomassie Brilliant Blue R-250. (C) Further purification of the MBP-MtRDR1 fusion protein by preparative gel filtration chromatography on a HiLoad Superdex 200 column (4ml extract volume loaded onto a column of volume 120ml) with elution in 50mM $NaH_2PO_4$, 0.15M NaCl pH 7.0 at a flow rate of 0.8ml/min. Fifty microliter samples from nine 1ml column fractions were analyzed by SDS-PAGE gel and protein revealed by staining with Coomassie Brilliant Blue R-250.
