# Supplementary material for: Salicylic acid treatment and expression of an RNA-dependent RNA polymerase 1 transgene inhibit lethal symptoms and meristem invasion during tobacco mosaic virus infection in Nicotiana benthamiana
Source: BMC Plant Biol. 2016 Jan 13;16:15. doi: 10.1186/s12870-016-0705-8 (PMC4710973; doi:10.1186/s12870-016-0705-8)
Supplement: Additional file 6: — Calibration curve to determine the molecular weight (log 10 MW) of protein detected by anti-MtRDR1 serum on SDS-PAGE gel. (PDF 72 kb) [file 12870_2016_705_MOESM6_ESM.pdf]

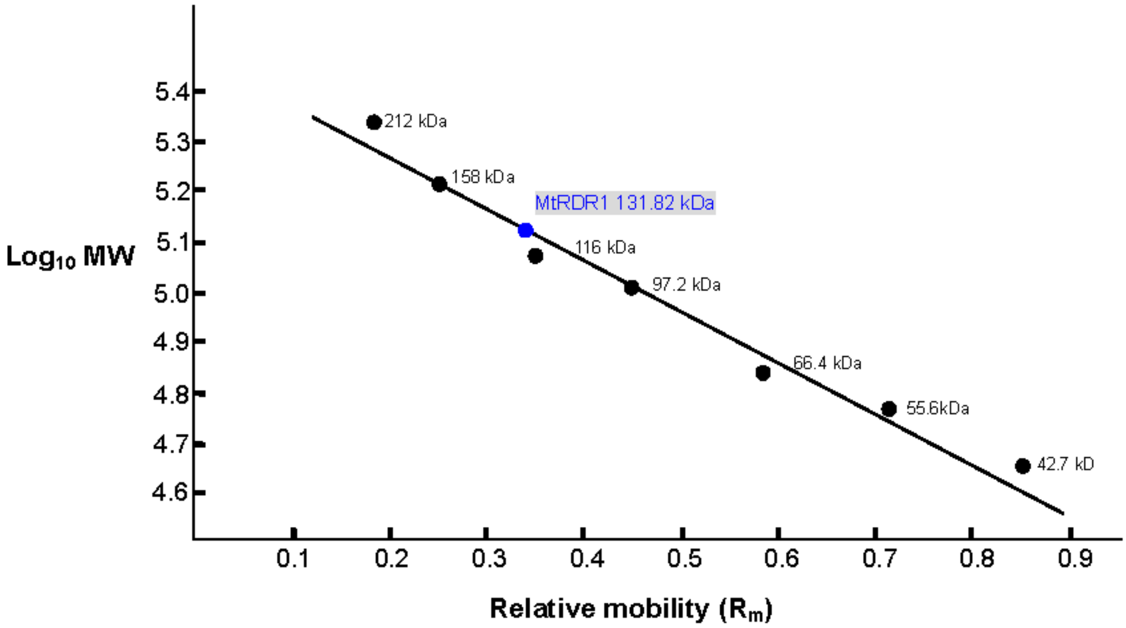

Additional File 6: Calibration curve to determine the molecular weight (log_{10} MW) of protein detected by anti-MtRDR1 serum on SDS-PAGE gel. A calibration curve of the log_{10} protein molecular weight (MW) versus relative mobility (R_m) was plotted for the molecular marker protein standards run on a 10% (w/v) acrylamide SDS-PAGE gel subjected to immunoblot analysis with anti-MtRDR1 serum. The protein standards used had relative molecular masses of 212, 158, 116, 97.2, 66.4, 55.6 and 42.7 kDa respectively. The curve was used to determine the molecular weight of the protein specifically detected by the anti-MtRDR1 serum as being 131.8 kDa, corresponding to MtRDR1.
