# Supplementary material for: Salicylic acid treatment and expression of an RNA-dependent RNA polymerase 1 transgene inhibit lethal symptoms and meristem invasion during tobacco mosaic virus infection in Nicotiana benthamiana
Source: BMC Plant Biol. 2016 Jan 13;16:15. doi: 10.1186/s12870-016-0705-8 (PMC4710973; doi:10.1186/s12870-016-0705-8)
Supplement: Additional file 7: — Predicted ubiquitination and phosphorylation sites in the MtRDR1 amino acid sequence. (PDF 153 kb) [file 12870_2016_705_MOESM7_ESM.pdf]

Predicted protein sequence for MtRDR1

MVKSKTIELYGFPHYVTVPDVKRFVEQYTGEGSVVAIKIREGKARIRTAFAIIQFTTKDHV
TDMMALPSRSLSPLQYGSSYLKVREMERDIVRRPREILHSLNDVKLYFCCQISEERLSV
LWREVDVRVDFGIGMRKWPFTLCHDNKTFKLELSYENIWKIELHQPRSKTTKYLLIQLIG
APRVFELYSPTSDDAYNVYEDLSKNYFSDPPDDQWIRAVDFTPCSCIGQSSALCLELPS
NRDFPNFRENFAQYEESAGPYMLESGSPFSCNLDVVPMVSPPQGIQIPFDILFKVNSLV
QHGCLSGSELDNAFYHLVDPTRVNVELIENALEKMYYSNDFCYEPVRWLKDQYKMYH
SSNNLPRSPTISLDNGLVYVRRAQITPCKVYFCGPEINVSNRVLRKYKKHMDNFLRVSF
VDEELDKLYSSDLSSRISVGGRTDIYYRILSILRNGIVIGDKKFEFLAFSSSQLRESSLWM
FARTPTGDTAGSIREWMGDFSRIKNVAKYAARLGQSFGSSTETTTVSSTEIEIILDVHNK
TKYVFSDGIGKISLELARRVAQKCGYDSMPSAFQIRYGGYKGVVAVDPTSSVKLSLRMS
MLKYDSDNTDLDVLACSKFRPCYLNRQLITLLSTLDVEDSVFEKKQKEAVDQLNTILTDS
TKAHEVLDLMSSGEVTNILKEMLICGYKPNVEPFLSMMLQTFRASKLLELRQKTRIFIPK
GRAMMGVLDETRTLEYGEVFVQYSNNREDSLSHVVNGKVVVAKNPCLYPGDVRVLKA
VDVPALRHMVDCVVFPQKGHRPHPNECSGSDLDGDIYFVCWDSELIPPKTIDPMEYDS
AKPIVLDHDVEIEEVQKYFTDYILNDCLGIIANAHTVFADKEPGKAMADPCVELAKLFSTA
VDFPKTGIPAEIPRGLFPTEYPDFMEKPDKPIYKSKNVIGKLFREIQGISTKDGSITSFTLE
VAKESYDSDMEFEGFMDYVDDAFYHKTNYDYKLGNLMDYYGIKTEAEILSGNIMKMSK
SFTKKRDADAITMAVRSLRKEARSWFTDSGAGVDSGSDDAYAKASAWYYVTYHHSYY
GLYNEGMQRDHFLSFPWCVYHLLVQIKKEKARMKMHSRMEQSFSHRLRLD

Additional File 7: Predicted ubiquitination and phosphorylation sites in the MtRDR1 amino acid sequence. Potential ubiquitination sites are back-shaded in black (medium confidence prediction) or high-lighted in green (low confidence prediction). Amino acid residues predicted to be potential phosphorylation sites in the translated MtRDR1 protein sequence are underlined and highlighted in blue. Phosphorylation sites were predicted using NetPhos 2.0 software [64]. Only residues with a potential phosphorylation score > 0.850 (high confidence prediction) have been marked above. Ubiquitination sites were predicted using UbPred software [65].
