# Supplementary material for: Salicylic acid treatment and expression of an RNA-dependent RNA polymerase 1 transgene inhibit lethal symptoms and meristem invasion during tobacco mosaic virus infection in Nicotiana benthamiana
Source: BMC Plant Biol. 2016 Jan 13;16:15. doi: 10.1186/s12870-016-0705-8 (PMC4710973; doi:10.1186/s12870-016-0705-8)
Supplement: Additional file 8: — Table of primers used for semi-quantitative and quantitative PCR. (PDF 76 kb) [file 12870_2016_705_MOESM8_ESM.pdf]

Additional File 8: Table of primers used for semi-quantitative and quantitative PCR

Target transcript	Primer name	Strand	Primer sequences (5' →3')	PCR Application
NbPR1a	PR1a 5'	F	AGGGATCCATGGGATTTGTTCTCTTT	Semi-Quantitative
	PR1a 3'	R	AGGAGCTCTTAGTATGGACTTTCGCC	
MtRDR1	MtRDR1 5'	F	ATTCCCTCATTATGTGACTGTGCC	Semi-Quantitative
	MtRDR1 3'	R	CCGTGGTTGATGCAGCTCAATC	
NbRDR1m	NbRDR1m 5'	F	TGTTGGGATCCAGACCTGGTT	Semi-Quantitative
	NbRDR1m 3'	R	TCAACTTCCTCAATTGTGACATCAT	
NbEF1α	EF1α 5'	F	ACGCTTGAGATCCTTAACCGCAACATTCTT	Semi-Quantitative
	EF1α 3'	R	TGGTGTCCTCAAGCCTGGTATGGTTGT	
MtRDR1	MtRDR1 QPCR A5'	F	AGGAGAAAGCCAGGATGAAGATGC	Quantitative
	MtRDR1 QPCR A3'	R	CAAGACGCAACCTGTGACTGAAAC	
NbEF1α	NbEF1α QPCR B5'	F	TGCCTTGTGGAAGTTTGAGACC	Quantitative
	NbEF1α QPCR B3'	R	GGTGGAGTCAATAATCAGGACAGC	
